# Unplanned visits and midwife-led antenatal care

**DOI:** 10.18332/ejm/157160

**Published:** 2022-12-22

**Authors:** Céleste M. Vincent, Loukia Μ. Spineli, Patricia Barlow, Mechthild M. Gross

**Affiliations:** 1 Midwifery Research and Education Unit, Hannover Medical School, Hannover, Germany; 2 Department of Obstetrics, Centre Hospitalier Universitaire Saint-Pierre, Brussels, Belgium

**Keywords:** prenatal care, after hours care, health services accessibility, midwifery, pregnancy classification

## Abstract

**INTRODUCTION:**

Midwives provide antenatal care to women to ensure the health of both mother and baby, according to women’s needs. This study aims to investigate demographic and social, clinical and obstetrical factors that may be associated with unplanned visits to the emergency by nulliparous and multiparous women who received midwifery care during the antenatal period.

**METHODS:**

This was a retrospective cohort study with data collection from medical records of the CHU Saint-Pierre hospital. A total of 971 women gave birth between 1 January and 31 December 2017 and received midwifery-led care during their pregnancy. Descriptive statistics and multivariable logistic regression models with 95% confidence intervals (95% CI) were performed separately for nulliparous and multiparous women.

**RESULTS:**

For nulliparae (n=246), the odds of visiting emergency services during pregnancy were 1.45 times (95% CI: 1.08–2.27) higher in women with more previous pregnancies than women with less previous pregnancies, 3.57 times (95% CI: 1.43–11.11) more likely in women without than with high-level hypertension, and 1.09 times (95% CI: 1.01–1.25) more likely in women with less previous midwifery-led visits than women with more previous midwifery-led visits. For multiparae (n=444), the odds of visiting emergency services during pregnancy were 2.12 times (95% CI: 1.06–6.07) higher in women presenting risk factors at first consultation than women without such factors.

**CONCLUSIONS:**

For nulliparous and multiparous women, some characteristics seem to be associated with unplanned visits. Spontaneous visits may be driven by a need for care perceived by women and/or their partner but not specifically by urgent or unfavorable medical conditions.

## INTRODUCTION

A major goal of antenatal care (ANC) is to ensure and maintain health for the mother and her unborn child by offering screening and providing social, mental and medical intervention when necessary^1^. This includes preventive and supportive care that helps women approach pregnancy and birth as positive experiences^2^, strengthens women’s capabilities, is fully suited to their needs^3^, and promotes normal reproductive processes. Moreover, according to Renfrew et al.^4^, ANC involves ‘first-line management of complications and accessible emergency treatment are provided when needed’. A NICE guideline examines intrapartum care for women with an existing medical condition or obstetric complications and their babies^5^. Midwifery is crucial to this approach, which requires effective interdisciplinary teamwork and integration across healthcare settings^4^.

In midwifery-led models, the midwife is the lead professional in caring for women during normal pregnancy and birth^6,7^, and this role is recognized internationally^2,8^. According to the Quality Maternal and Newborn Care framework (QMNC framework), different models of ANC have been shown to improve maternal and neonatal outcomes^9^. The taxonomy presented by Symon et al.^7^ identifies various antenatal models of care where midwifery-led care provides effective and beneficial interventions. However, while some outcomes have been shown to be improved, there is uncertainty about why certain models of care are more effective compared to others^9^. More health-oriented research rather than a risk-based approach is needed^10^ to investigate further the mechanisms and the potentials of midwifery-led care as an intervention, as studied by Symon et al.^7^.

In addition to ANC for normal pregnancies, midwives also care for women who have factors that require special attention^4^. The huge increase in midwifery care of pregnant women with social factors is challenging for pregnant women to avoid being labelled as ‘not low-risk’^7^. Indeed, women living in very precarious situations without health insurance or financial resources, present more factors associated with poor perinatal outcomes^1,11,12^. However, clear and complete definitions of those complications and the associated clinical and social risk factors are not available. Consequently, the scaling for the level of care according to those factors is ambiguous. Constant and continuous assessment of factors is an area of interest for midwifery care, fulfilling the full scope of practice^4^. These aspects of midwifery care − which require additional monitoring − lead to medical consultation referrals when high-level complications arise or are detected^13^. Some countries have developed national guidelines detailing conditions and categories for referral^14,15^; however, no classification terminology of risk factors and referral levels is currently available, to our knowledge.

In this study, we focused on a salutogenic approach^10^. Indeed, perinatal care research and practice often focus more on interventions that minimize or prevent adverse health outcomes than on interventions that reinforce health and wellbeing^10^. Women need healthcare systems that support them to stay healthy and that provide a timely transition to elective and emergency care when a complication occurs^4^. A British analysis showed that the most socially disadvantaged women were 60% less likely to have received any ANC when compared to the least socially disadvantaged women^16^. Demographic and social factors (e.g. social disadvantages, marginalized population) and access to care (e.g. insurance coverage) may affect initial and subsequent sustained access to ANC^1,17^. These factors could partially explain why maternal and neonatal health inequalities remain in ANC^18^. Therefore, the use of unplanned visits to emergency services can be considered as an important access point to provide healthcare during pregnancy for women who might otherwise experience a lack of ANC or do not have access to ANC at all.

Visits of pregnant women to emergency services are unplanned visits outside of scheduled prenatal care^19^. Other studies refer to contacts^2^, out-of-hours visits^20^, and emergency department visits^21^. In hospital-based emergency settings, obstetricians, midwives and/or nurses provide permanent care 24 hours a day, seven days a week^22^. Worldwide, in low- and high-income countries, growth in the demand for general emergency healthcare services is observed, as confirmed by a report from the Organization for Economic Cooperation and Development (OECD)^22,23^ and maternity emergency healthcare is no exception.

A specific feature of emergency services during pregnancy is that women most often appear without life-threatening symptoms. However, a clear definition of ‘non-urgent’ cases (i.e. which are non-threatening or do not require immediate attention)^24^ or ‘false alarms’ is extremely difficult to provide because of the discrepancy between the perceptions of healthcare providers and users. In this respect, urgent and severe cases are distinct (e.g. perceived decrease of fetal movements, perceived onset of labor, vaginal bleeding). Besides, attempting to define ‘unjustified’, ‘inappropriate’, or ‘non-urgent’ reasons for a visit to emergency services, according to an efficiency-driven notion, is in contradiction with the promotion of the WHO guidelines that focus on a positive experience of pregnancy^2^, and with the women-centered care concept^3^.

Healthcare visits to emergency services can be considered as access to care when needed or perceived as needed. Indeed, women who do not attend their scheduled visits (e.g. women who are members of marginalized population groups^17^) have at least the opportunity of seeking emergency healthcare appointments. Insights into why women may not attend ANC or why they may attend once and then not again because of mistreatment in formal maternity care systems, have recently been recognized in a Cochrane review^25^. Our research adds empirical data to this important question. The availability of resources, including treatment, laboratory tests or ultrasound, as well as the access to medical facilities and specialist tests in one place, are all good reasons for women to use emergency healthcare services^22^. Unplanned visits can, in some cases, be seen as positive as they compensate for the inadequate use of fragmented ANC.

In the present study, we will investigate demographic, social, clinical and obstetrical factors that may be associated with unplanned visits to the emergency by pregnant nulliparous and multiparous women who received midwifery care during the antenatal period.

## METHODS

This retrospective cohort study took place at CHU Saint-Pierre, a tertiary hospital in Belgium. Due to the hospital’s geographical location and public status, a considerable proportion of women living in low socio-economic levels and migrant women are referred there. Midwifery-led ANC in the hospital includes scheduled visits. In this midwifery-led model of care, midwives are the lead healthcare professionals responsible for the planning, organization and delivery of care given to a woman with a normal pregnancy from the initial booking of antenatal visits through to care during the postnatal period in collaboration with obstetricians when needed. Continuity of care during the antenatal period is encouraged as much as possible but not guaranteed, and antenatal visits are usually scheduled with the same healthcare provider once the level of care has been determined at the first antenatal consultation.

‘Level 1’ of care offered is midwifery care for women with ‘low-risk’ pregnancy, that is, clinical and obstetrical conditions with no further examination required during pregnancy and with no particular guidelines applied regarding the progress of pregnancy and mode of birth. ‘Level 2’ of care is designated midwifery care requiring additional discussion or a consultation with an obstetrician or another specialist for women with ‘medium-risk’ pregnancy, that is, conditions requiring a discussion or a consultation by an obstetrician and/or another qualified and competent professional (e.g. gestational diabetes without insulin, chronic infections, previous cesarean, previous conization). ‘Level 3’ alludes to women with ‘high-risk’ pregnancy, when the situation required that the responsibility for the woman’s care should be transferred to an obstetrician. There was a need for new decisions on how to monitor the pregnancy, and the timing and mode of birth needed to be discussed with the woman, if possible (e.g. preeclampsia, cholestasis, multiple pregnancies). In those situations, obstetricians would assume ongoing clinical responsibility. Midwives assumed coordination of women’s care with the obstetricians allowing continuity of care and maintaining a trustful relationship with women.

At CHU Saint-Pierre, midwives oversee women with normal pregnancies, births and postnatal care autonomously, and women with ‘medium-risk’, in collaboration with obstetricians and other specialists. For normal pregnancies, monthly antenatal visits are performed from the first contact until around 32 completed weeks, every two weeks until 40 completed weeks, and then every week around 40 and 41 completed weeks. Regular surveillance for pregnancies that need further monitoring without hospitalization (e.g. cardiotocography, blood pressure check-up), and cardiotocography for pregnancies that passed their estimated date of delivery (between 40 and 42 completed weeks of gestation and beyond) are performed in the medical dispensary (‘dispensaire’). Appointments are scheduled in advance during pregnancy and last-minute antenatal visits on demand are less accessible. In emergency healthcare services for childbearing women at CHU Saint-Pierre, women visiting the emergency healthcare services are seen in the general accident and emergency department below 20 completed weeks of gestational age or in the obstetrical emergency department between 20 weeks and 42 weeks. When they come spontaneously, women go directly to the emergency departments, and triage takes place on-site, without prior telephone call or medical advice.

Ethical approval was obtained from the Hannover Medical School and the CHU Saint-Pierre hospital. This observational study did not create ethical issues regarding treatment allocation or data collection. Patient consent for sharing data was not required in Belgium at the time of data collection. No conflict of interest was reported. Visits to emergency healthcare services were presented on scanned paper and compiled manually in digital files (CHU Saint-Pierre, 2017).

To study specifically midwifery-led care as an intervention in the antenatal model of care, in a more health oriented rather than a risk-based approach^9,10^, women were selected when midwifery-led visits were performed at first visit or during pregnancy (n=971; median=7 and standard deviation equal to 2.8 and 2.9 for nulliparous and multiparous women, respectively). Women with ‘low-risk’, ‘medium-risk’ and ‘high-risk’ pregnancies were included if they attended a midwifery-led planned visit. Pregnant women who received exclusively obstetric-led ANC were not selected.

Only cases of spontaneous, non-scheduled visits to emergency departments for obstetrical reasons during pregnancy were included. Cases of pregnant women in active labor were excluded, as well as visits for reasons without direct relation to the pregnancy, requiring no specific obstetrical follow-up (e.g. dental infection, orthopedic issues), or visits in other healthcare settings, or visits referred by a healthcare provider.

Of the 3422 live births in 2017 in CHU Saint-Pierre hospital, 971 pregnant women fulfilled the inclusion criteria. Maternal age (in years)^1,9,26^, maternal nationality (subdivided into Belgium, EU except for Belgium, other countries)^1,17-19,26^ and maternal body mass index (BMI, in kg/m^2^) before pregnancy or early pregnancy^1,26^ were selected as demographic factors. Legal civil status (subdivided into married, single, divorced/widowed)^1^, actual civil status (subdivided into couple, family as support system, lives alone, home shelter/other) very precarious situation (homeless women, asylum seekers, undocumented residents, women from travelling communities) and without health insurance coverage^11,17,19^, education level (subdivided into primary, lower secondary, secondary superior, and post-secondary non-tertiary/other)^1^, and occupational situation (subdivided into employed, unemployed/social assistance, stay-at-home mother, and without stable income/student/other)^1^ were included as social factors. The precise income was not specified in the medical files, but the occupational situation could provide a rough estimate. All these variables were population characteristics, and factors that were core and 170 recommended maternal health indicators^1^.

Obstetrical history was assessed with respect to gravidity (continuously counted)^1,26^ and previous cesarean section or uterine surgery^26^. Obstetrical variables were singleton pregnancy^1,26^, suspected oligohydramnios, suspected macrosomia, suspected intrauterine growth restriction (IUGR), neonatal weight^1^ and gestational age at birth^1^, the latter to provide information on fetal/neonatal health. Neonatal weight (kg) and gestational age at birth (days) were selected as pregnancy variables since these variables provide more accurate information on fetal/neonatal outcomes than suspected macrosomia, suspected intrauterine growth, or premature birth threat. Regarding clinical characteristics, the following variables have been retrieved from the database: hypertension (blood pressure: medium-risk level >140/90 mmHg; high-risk level >160/110 mmHg), gestational diabetes (medium level: without insulin treatment during pregnancy; high level: pre-existing before pregnancy and/or with insulin treatment during pregnancy), thyroid disease, serological status for HIV (human immunodeficiency virus), and obesity during pregnancy (BMI ≥30)^1,26^. The variables (multiple births, maternal age, parity, and maternal pre-pregnancy body mass index) describe characteristics of the pregnant women that are related to risks of mortality, morbidity, and obstetric interventions^1^. The other variables were indicators of fetal/neonatal health, maternal health, maternal characteristics and risk factors, and health services^1^. Characteristics of variables are presented in the Supplementary file.

Antenatal care was presented as level of care (i.e. presence or absence of ‘high-risk’ factors identified at the first consultation and during pregnancy, and hospitalization during pregnancy), adequacy of ANC (i.e. number of scheduled antenatal visits, gestational age at the first visit, the total number of midwifery-led visits) and the number of visits to emergency healthcare services. According to the Belgian Health Care Knowledge Centre, ten and seven antenatal visits are recommended for nulliparous and multiparous women without risk factors, respectively^27^.

To avoid substantial listwise deletion that comes with analyzing variables with considerable missing data, which in turn may compromise the validity of results if the analyzed sample (after listwise deletion) differs systematically from the original sample, variables with missing data above 30% were not analysed^28^.

Obstetrical characteristics for current pregnancy were extracted from the database. An external reviewer double-checked 10% of the dataset (n=97 cases). Identification numbers were retrieved for every 10 cases to guarantee systematic sampling. Double entry was applied afterwards for data checking^29^. Once the collection had been performed, data were compared to the initial data collected.

Characteristics were summarized descriptively for women who visited and those who did not visit emergency services. Specifically, absolute and relative frequencies were used for categorical variables, whilst measures of central tendency (mean, median, and mode) and dispersion (standard deviation, interquartile range, and range) were provided for continuous variables. The descriptive analysis revealed that some of the categorical variables had very low frequencies, especially the binary ones (i.e. multiple pregnancies, suspect oligoamnios, suspected IUGR). For these variables, the standard errors of log odds ratio were implausibly large, indicating a situation of separation where a single independent variable or a linear combination of independent variables predicts perfectly the binary outcome^29^.

Descriptive statistics were performed using the statistical software Statistical Package for the Social Science (SPSS) Version 25. To investigate predictors of one or more visits to emergency services (yes/no), we conducted a multivariable logistic regression separately for nulliparous and multiparous women (the former was about to give birth for the first time, whereas the latter already gave birth at least once), adjusting for the same demographic, social, obstetrical, clinical and antenatal-care characteristics. We encountered separation issues with categorical variables with less than 1% relative frequency in at least one level. We partially addressed the separation issue using Firth’s bias reduction method^30,31^ in each multivariable logistic regression. We used the R-package logistf^30^ to implement Firth’s bias reduction method (R software, version 3.5.1). Model convergence persisted after implementing the Firth’s bias reduction method. To ensure model convergence, we merged levels with total counts <1%; for instance, in the occupational situation (five levels), we merged ‘student/other’ with ‘without stable income’. In the multivariable logistic regression model for nulliparous women, we did not include the binary variables ‘serological status HIV positive’ and ‘previous cesarean or uterine surgery’, since <1% of the women represented these variables, and consequently, the model could not converge. We did not perform multiple imputation to address missing data in the independent variables. We did not use any stepwise procedure to build the multivariable logistic regression model as we referred to relevant published literature to decide on the clinically important characteristics for our study.

## RESULTS

In the sample (n=971), 36% (n=348) of women were nulliparous, and 64% (n=623) were multiparous (median: 2 children; range: 0–13). More than half (54%) of all the women had at least one spontaneous visit to emergency healthcare services. In the sample (n=971), the mean number of visits to emergency healthcare services was around one with a wide distribution (nulliparae: 1–11, multiparae: 1–12).

Overall, nulliparous women were slightly younger compared to multiparous women (range: 14–44 years, in both groups) regardless of the visit to emergency services (Table 1). Overall, 70 nationalities from around the world were represented; around one in three women were of Belgium nationality and <20% were from European Union countries, whereas more than half of the women were of non-European nationality regardless of parity and visit to emergency services. Median BMI (kg/m^2^) was slightly larger in multiparous women but similar between those with and those without visits to emergency services (nulliparae: 20 without and 24 with visits to emergency services; multiparae: 26 without and 25 with visits to emergency services). Most of the nulliparous women were single (55%), whereas most of the multiparous women were married (63%), irrespective of visits to emergency services. The great majority of women lived as a couple; around two in five nulliparous women regardless of visits to emergency services, where 50% and 91% multiparous women with and without visits to emergency services, respectively.

**Table 1:** Demographic and social characteristics for nulliparous and multiparous women who visited or not the emergency services, Belgium 2018 (N=971)

*Characteristics*	*Nulliparous*		*Multiparous*	
	*Visits to emergency services*			
	*No (n=142)*	*Yes (n=206)*	*No (n=305)*	*Yes (n=318)*
**Demographic**				
**Maternal age** (years)				
Median (IQR)	27.0 (23.0–32.0)	26.0 (22.0–30.0)	31.0 (27.0–35.0)	31.0 (27.0–34.3)
Mean (SD)	27.7 (5.7)	26.3 (5.6)	31.2 (5.4)	30.6 (5.4)
Mode (range)	23 (17–44)	26 (14–44)	29 (19–43)	32 (17–44)
Missing	0 (0.0)	0 (0.0)	0 (0.0)	0 (0.0)
**Maternal nationality** (%)				
Belgium	43 (30.5)	69 (34.2)	88 (29.2)	71 (22.5)
EU, except for Belgium	28 (19.9)	28 (13.9)	35 (11.6)	42 (13.3)
Other	70 (49.6)	105 (52.0)	178 (59.1)	202 (64.1)
Missing	1 (0.7)	4 (1.9)	4 (1.3)	3 (0.9)
**BMI before pregnancy** (kg/m^2^)				
Median (IQR)	20.0 (20.8–27.3)	24.3 (20.3–27.2)	26.1 (22.1–29.0)	25.2 (22.6–28.7)
Mean (SD)	24.2 (4.1)	24.8 (5.0)	26.2 (4.8)	26.0 (5.1)
Mode (range)	20.0 (15.6–36.4)	24.1 (16.7–52.0)	22.0 (16.1–41.1)	23.4 (15.5–46.5)
Missing	17 (12.0)	22 (10.7)	43 (14.1)	38 (11.9)
**Legal civil status** (%)				
Married	63 (46.0)	84 (41.8)	204 (68.9)	172 (57.0)
Single	73 (53.3)	113 (56.2)	80 (27.0)	118 (39.1)
Divorced/widowed	1 (0.7)	4 (2.0)	12 (4.1)	12 (4.0)
Missing	5 (3.5)	5 (2.4)	9 (3.0)	16 (5.0)
**Actual civil status** (%)				
Couple	116 (84.7)	168 (83.6)	269 (90.9)	268 (88.7)
Family (support system)	7 (5.1)	16 (8.0)	5 (1.7)	8 (2.6)
Lives alone	9 (6.6)	11 (5.5)	13 (4.4)	19 (6.3)
Home shelter/other	5 (3.6)	6 (3.0)	9 (3.0)	7 (2.3)
Missing	5 (3.5)	5 (2.4)	9 (3.0)	16 (5.0)
**Social**				
**Very precarious situation** (%)				
Yes	37 (26.1)	44 (21.4)	41 (13.4)	37 (11.6)
No	105 (73.9)	162 (78.6)	264 (86.6)	281 (88.4)
Missing	0 (0.0)	0 (0.0)	0 (0.0)	0 (0.0)
**Education level** (%)				
Primary	29 (22.3)	50 (25.6)	95 (31.3)	91 (31.3)
Lower secondary	23 (17.7)	36 (18.5)	65 (22.9)	54 (18.6)
Secondary superior	38 (29.2)	52 (26.7)	83 (29.2)	99 (34.0)
Post-secondary non-tertiary/other	40 (30.8)	57 (29.2)	41 (14.4)	47 (16.1)
Missing	12 (8.5)	11 (5.3)	21 (6.9)	27 (8.5)
**Occupational situation** (%)				
Employed	48 (35.0)	67 (33.3)	63 (21.4)	73 (24.3)
Unemployed/social assistance	26 (19.0)	40 (19.9)	79 (26.8)	85 (28.3)
Stay-at-home mother	19 (13.9)	21 (10.4)	94 (31.9)	76 (25.3)
Without stable income/student/other	44 (32.1)	73 (36.3)	59 (20.0)	66 (22.0)
Missing	5 (3.5)	5 (2.4)	10 (3.3)	18 (5.7)

IQR: interquartile range. SD: standard deviation. BMI: body mass index.

Overall, 23% of nulliparous and 13% of multiparous women lived in a very precarious situation. Among women visiting emergency services, only 8% and 7% were, respectively, nulliparous and multiparous women living in very precarious situation (Table 1). Among nulliparous women who visited emergency services, the majority had a post-secondary non-tertiary or another education level (29%) and had no stable income, followed by the student or other occupational situations (36%) or employed (33%). In contrast, among multiparous women who visited emergency services, the majority had a secondary superior education level (34%) and were unemployed or received social assistance (28%), or were stay-at-home mothers (25%).

Overall, gravidity was widely distributed, ranging 1–15. Around 20% of women had a previous cesarean section or previous uterine surgery among multiparous women, regardless of visits to emergency services (Table 2). Generally, the distribution of neonatal birth weight was quite wide, ranging 0.480–4.700 kg. Multiparous women had babies with a larger mean neonatal weight than nulliparous women. Less than 1% of newborns were born extremely preterm or very preterm (before 28 completed weeks or from 28 to 32 completed weeks, respectively), whereas 4% were moderate to late preterm (32 to 37 completed weeks). Most newborns were born at term, after 37 completed weeks of gestational age (96%).

**Table 2:** Obstetrical characteristics for nulliparous and multiparous women who visited or not the emergency services, Belgium 2018 (N=971)

*Characteristics*	*Nulliparous*		*Multiparous*	
	*Visits to emergency services*			
	*No (n=142)*	*Yes (n=206)*	*No (n=305)*	*Yes (n=318)*
**History**				
**Gravidity**				
Median (IQR)	1.0 (1.0–2.0)	1.0 (1.0–2.0)	3.0 (2.0–4.0)	3.0 (2.0–4.0)
Mean (SD)	1.3 (0.6)	1.4 (0.7)	3.5 (1.6)	3.5 (1.6)
Mode (range)	1 (1–4)	1 (1–5)	2 (2–10)	2 (2–15)
Missing	0 (0.0)	0 (0.0)	0 (0.0)	0 (0.0)
**Previous cesarean or uterine surgery** (%)				
Yes	0 (0.0)	1 (0.6)	65 (22.3)	54 (17.8)
No	109 (100.0)	156 (99.4)	227 (77.7)	249 (82.2)
Missing	33 (23.2)	49 (23.8)	13 (4.3)	15 (4.7)
**Current pregnancy**				
**Singleton pregnancy** (%)				
Yes	140 (98.6)	205 (99.5)	304 (99.7)	316 (99.4)
No	2 (1.4)	1 (0.5)	1 (0.3)	2 (0.6)
Missing	0 (0.0)	0 (0.0)	0 (0.0)	0 (0.0)
**Suspect oligoamnios** (%)				
Yes	1 (0.7)	1 (0.5)	3 (1.0)	2 (0.6)
No	141 (99.3)	205 (99.5)	302 (99.0)	316 (99.4)
Missing	0 (0.0)	0 (0.0)	0 (0.0)	0 (0.0)
**Suspected macrosomia** (%)				
Yes	11 (7.7)	15 (7.3)	28 (9.2)	32 (10.1)
No	131 (92.3)	191 (92.7)	277 (90.8)	286 (89.9)
Missing	0 (0.0)	0 (0.0)	0 (0.0)	0 (0.0)
**Suspected IUGR** (%)				
Yes	7 (4.9)	2 (1.0)	3 (1.0)	2 (0.9)
No	135 (95.1)	204 (99.0)	302 (99.0)	315 (99.1)
Missing	0 (0.0)	0 (0.0)	0 (0.0)	0 (0.0)
**Neonatal weight** (kg)				
Median (IQR)	3.2 (2.9–3.6)	3.2 (3.0–3.6)	3.3 (3.0–3.7)	3.3 (3.0–3.7)
Mean (SD)	3.2 (0.6)	3.3 (0.5)	3.3 (0.5)	3.3 (0.5)
Mode (range)	3.2 (0.5–4.2)	3.1 (1.8–4.7)	3.2 (0.7–4.6)	3.3 (1.3–4.6)
Missing	0 (0.0)	0 (0.0)	0 (0.0)	0 (0.0)
**Gestational age at birth** (days)				
Median (IQR)	278.0 (272.0–284.0)	280.0 (273.8–285.0)	279.0 (272.0–285.0)	278.0 (272.0–283.0)
Mean (SD)	275.1 (16.6)	280.0 (10.7)	276.9 (13.0)	276.8 (9.7)
Mode (range)	289 (176–291)	283 (229–294)	278 (175–192)	280 (213–294)
Missing	0 (0.0)	0 (0.0)	0 (0.0)	0 (0.0)

IQR: interquartile range. IUGR: intrauterine growth restriction. SD: standard deviation.

Hypertension was observed in 2% of women, with 3% and 2% of nulliparous and multiparous women, respectively, having medium-level hypertension (Table 3). Furthermore, about two in ten women had gestational diabetes without insulin. Among nulliparous women, 17% were diagnosed with gestational diabetes without insulin treatment required, as opposed to 22% among multiparous women. Moreover, 9% of women had hypothyroidism regardless of parity. Overall, 17% of the women were identified as obese during pregnancy; 14% and 19% had obesity during pregnancy among nulliparous and multiparous women, respectively. Among those women who visited emergency services, 15% of nulliparous were obese compared to 18% of multiparous.

**Table 3:** Clinical and antenatal care characteristics for nulliparous and multiparous women who visited or not the emergency services, Belgium 2018 (N=971)

*Characteristics*	*Nulliparous*		*Multiparous*	
	*Visits to emergency services*			
	*No (n=142)*	*Yes (n=206)*	*No (n=305)*	*Yes (n=318)*
**Clinical**				
**Hypertension** (%)				
Medium-level	2 (1.4)	7 (3.4)	7 (2.3)	5 (1.6)
High-level	10 (7.0)	3 (1.5)	5 (1.6)	6 (1.9)
No	130 (91.5)	196 (95.1)	293 (96.1)	307 (96.5)
Missing	0 (0.0)	0 (0.0)	0 (0.0)	0 (0.0)
**Gestational diabetes** (%)				
Medium-level	30 (21.1)	29 (14.1)	77 (25.2)	60 (18.9)
High-level	4 (2.8)	9 (4.4)	8 (2.6)	15 (4.7)
No	108 (76.1)	168 (81.6)	220 (72.1)	243 (76.4)
Missing	0 (0.0)	0 (0.0)	0 (0.0)	0 (0.0)
**Thyroid disease** (%)				
Yes	13 (9.8)	21 (10.2)	32 (10.5)	33 (10.4)
No	128 (90.1)	185 (89.8)	273 (89.5)	285 (89.6)
Missing	0 (0.0)	0 (0.0)	0 (0.0)	0 (0.0)
**Serological status HIV positive** (%)				
Yes	0 (0.0)	1 (0.5)	2 (0.7)	0 (0.0)
No	142 (100.0)	205 (99.5)	303 (99.3)	318 (100.0)
Missing	0 (0.0)	0 (0.0)	0 (0.0)	0 (0.0)
**Obesity during pregnancy** (%)				
Yes	16 (11.3)	31 (15.0)	59 (19.3)	58 (18.2)
No	126 (88.7)	175 (85.0)	246 (80.7)	260 (81.8)
Missing	0 (0.0)	0 (0.0)	0 (0.0)	0 (0.0)
**Antenatal care**				
**High-risk factors at first consultation** (%)				
Yes	6 (4.2)	12 (5.8)	38 (12.5)	56 (17.6)
No	136 (95.8)	194 (94.2)	267 (87.5)	262 (82.4)
Missing	0 (0.0)	0 (0.0)	0 (0.0)	0 (0.0)
**High-risk factors during pregnancy** (%)				
Yes	19 (13.4)	24 (11.7)	33 (10.8)	50 (15.7)
No	123 (86.6)	182 (88.3)	272 (89.2)	268 (84.3)
Missing	0 (0.0)	0 (0.0)	0 (0.0)	0 (0.0)
**Hospitalization during pregnancy** (%)				
Yes	7 (4.9)	25 (12.1)	10 (3.3)	53 (16.7)
No	135 (95.1)	181 (87.9)	295 (96.7)	265 (83.3)
Missing	0 (0.0)	0 (0.0)	0 (0.0)	0 (0.0)
**Adequate antenatal care** (%)				
Yes	43 (35.2)	79 (41.8)	222 (77.6)	249 (82.7)
No	79 (64.8)	110 (58.2)	64 (22.4)	52 (17.3)
Missing	20 (14.1)	17 (8.3)	19 (6.2)	17 (5.3)
**Gestational age at first consultation** (%)				
20 weeks and before	126 (92.0)	185 (93.9)	259 (86.9)	287 (92.9)
After 20 weeks	11 (8.0)	12 (6.1)	39 (13.1)	22 (7.1)
Missing	5 (3.5)	9 (4.4)	7 (2.3)	9 (2.8)
**Total number of midwifery-led visits**				
Median (IQR)	7.0 (5.0–8.6)	7.0 (5.0–9.0)	6.0 (4.0)	7.0 (4.0–8.0)
Mean (SD)	6.5 (2.8)	6.5 (2.7)	6.1 (3.0)	6.0 (2.9)
Mode (range)	8 (1–12)	9 (1–12)	7 (1–14)	8 (1–13)
Missing	0 (0.0)	0 (0.0)	0 (0.0)	0 (0.0)
**Total number of spontaneous visits**				
Median (IQR)	0.0 (0.0)	2.0 (1.0–3.0)	0.0 (0.0)	1.0 (1.0–2.0)
Mean (SD)	0.0 (0.0)	2.2 (1.6)	0.0 (0.0)	1.9 (1.5)
Mode (range)	0 (0–0)	1 (1–11)	0 (0–0)	1 (1–12)
Missing	0 (0.0)	0 (0.0)	0 (0.0)	0 (0.0)
**Total number of visits to AED**				
Median (IQR)	0.0 (0.0)	0.0 (0.0–1.0)	0.0 (0.0)	0.0 (0.0–1.0)
Mean (SD)	0.0 (0.0)	0.5 (0.8)	0.0 (0.0)	0.6 (0.8)
Mode (range)	0 (0–0)	0 (0–4)	0 (0–0)	0 (0–7)
Missing	0 (0.0)	0 (0.0)	0 (0.0)	0 (0.0)
**Total number of visits in OED**				
Median (IQR)	0.0 (0.0)	1.0 (1.0–2.0)	0.0 (0.0)	1.0 (1.0–2.0)
Mean (SD)	0.0 (0.0)	1.6 (1.5)	0.0 (0.0)	1.4 (1.3)
Mode (range)	0 (0–0)	1 (0–9)	0 (0–0)	1 (0–8)
Missing	0 (0.0)	0 (0.0)	0 (0.0)	0 (0.0)

AED: accident and emergency department. IQR: interquartile range. OED: obstetrical emergency department. SD: standard deviation.

Overall, according to the criteria established at CHU Saint-Pierre hospital, 88% of women had no risk factors identified at the first consultation. Among nulliparous women who visited emergency services, 94% had no risk factors identified at their first antenatal consultation compared to 82% among multiparous women who visited emergency services (Table 3). Eighty-seven and eighty-nine per cent of women had ‘low’ or ‘medium-risk’ factors at first antenatal consultation and during pregnancy, respectively. Ninety per cent of women were never hospitalized during their pregnancy. Eleven per cent of women presented ‘high-risk’ factors at the first consultation. Thirteen per cent of women had ‘high-risk’ factors that occurred during pregnancy, requiring referral to an obstetrician according to the hospital’s protocol. Ten per cent of women were hospitalized during pregnancy.

Only 61% of women had adequate ANC, with 39% and 80% of nulliparous and multiparous women, respectively, having more than ten planned antenatal visits and more than seven planned antenatal visits (Table 3). Among those women who did not visit emergency services, 65% of nulliparous women did not attend an adequate number of scheduled antenatal visits, compared to 22% of multiparous women. Overall, 9% had a late first consultation (after 20 weeks of gestational age). Among those women who did not visit emergency services, 8% of nulliparous had a late ANC initiation, compared to 13% of multiparous women. The mean of the total number of midwifery-led antenatal scheduled visits was around six visits. Specifically, the total number of midwifery-led antenatal scheduled visits ranged from one to 12 or 14 in nulliparous and multiparous women, respectively. Nulliparous women had a larger median number of midwifery-led visits than multiparous women.

Regarding the BMI before pregnancy, 11% of cases had missing data among nulliparous women and 13% among multiparous women, with more missing data cases in both ‘no visit to emergency services’ (Table 3). In the group of nulliparous women who never came to emergency services, the missing rate was the highest (14%) regarding the adequate number of antenatal scheduled visits compared to other groups.

In the multivariable logistic regression model, regardless of parity, demographic and social characteristics did not appear to predict visits to emergency services compared to those who had none (Table 4). For nulliparous women, the odds of visiting emergency services at least once during pregnancy were 1.45 times (95% CI: 1.08–2.27) more likely in nulliparous women with more previous pregnancies (i.e. including abortion or miscarriage) than nulliparous women with less previous pregnancies (Table 4). Regarding clinical characteristics, for nulliparous women, the odds of visiting emergency services during pregnancy were 3.57 times (95% CI: 1.43–11.11) more likely in women without than with high-level hypertension. In both parity groups, other clinical characteristics did not appear to predict emergency service visits. Regarding ANC, for nulliparous women, the odds of visiting emergency services during pregnancy were 1.09 times (95% CI: 1.01–1.25) more likely in women with less previous midwifery-led visits compared to women with more previous midwifery-led visits. For multiparous women, the odds of visiting emergency services during pregnancy were 2.12 times (95% CI: 1.06–6.07) more likely in women presenting factors associated with ‘high-risk’ factors at first consultation compared to women without such factors.

**Table 4:** Multivariable logistic regression results per parity, Belgium 2018 (N=971)

*Variables*	*Nulliparous AOR (95% CI)*	*Multiparous AOR (95% CI)*
**Demographic**		
**Maternal age**	0.97 (0.92–1.01)	0.98 (0.93–1.03)
**Maternal nationality**		
EU, except for Belgium	0.77 (0.40–1.41)	1.24 (0.57–3.14)
Other	1.05 (0.58–2.00)	1.26 (0.72–2.26)
Belgium (Ref.)	1	1
**BMI before pregnancy**	1.05 (0.99–1.14)	1.02 (0.96–1.09)
**Legal civil status**		
Single	1.43 (0.89–2.57)	1.38 (0.80–2.64)
Divorced/widowed	12.93 (1.36–47228.49)	0.78 (0.29-2.42)
Married (Ref.)	1	1
**Actual civil status**		
Family	1.00 (0.37–3.17)	1.55 (0.37–21.99)
Lives alone	0.72 (0.28–2.01)	1.40 (0.44–10.25)
Home-shelter/other	0.51 (0.16–1.67)	0.41 (0.10–1.93)
Couple (Ref.)	1	1
**Social**		
**Very precarious situation**		
Yes	0.70 (0.35–1.22)	0.72 (0.28–1.76)
No (Ref.)	1	1
**Education level**		
Lower secondary	0.64 (0.29–1.20)	1.09 (0.59–2.09)
Secondary superior	1.21 (0.66–2.39)	1.31 (0.74–2.51)
Post-secondary non-tertiary or other	1.26 (0.68–2.54)	1.02 (0.49–2.17)
Primary (Ref.)	1	1
**Occupational situation**		
Unemployed/social assistance	1.14 (0.58–2.44)	0.62 (0.29–1.18)
Stay-at-home mother without social welfare	1.10 (0.50–2.51)	0.64 (0.30–1.20)
No stable income or student/other	1.76 (0.92–3.99)	0.98 (0.40–2.55)
Employed (Ref.)	1	1
**Obstetrical**		
**Gravidity**	1.45 (1.08–2.27)	1.00 (0.87–1.18)
**Previous cesarean**		
Yes		0.75 (0.41–1.32)
No (Ref.)		1
**Singleton pregnancy**		
Yes	0.34 (0.00–1470.60)	0.61 (0.01–1918.89)
No (Ref.)	1	1
**Suspect oligoamnios**		
Yes	17.52 (1.23–1685.24)	0.68 (0.05–2137.81)
No (Ref.)	1	1
**Suspected macrosomia**		
Yes	1.05 (0.43–2.99)	1.30 (0.60–3.21)
No (Ref.)	1	1
**Suspected IUGR**		
Yes	0.08 (0.00–0.33)	0.89 (0.12–15.40)
No (Ref.)	1	1
**Neonatal weight** (kg)	1.34 (0.78–2.56)	0.81 (0.42–1.53)
**Gestational age at birth** (days)	1.01 (0.99–1.05)	1.02 (0.99–1.05)
**Clinical**		
**Hypertension**		
Medium-level	10.45 (0.44–41267.40)	0.70 (0.17–4.69)
High-level	0.28 (0.09–0.70)	0.80 (0.08–15.27)
No (Ref.)	1	
**Gestational diabetes**		
Medium-level	0.61 (0.33–1.01)	0.71 (0.41–1.18)
High-level	1.49 (0.44–11.74)	1.50 (0.47–10.51)
No (Ref.)	1	1
**Thyroid disease**		
Yes	1.26 (0.62–3.31)	1.00 (0.49–2.36)
No (Ref.)	1	1
**Obesity during pregnancy**		
Yes	0.53 (0.20–1.13)	0.73 (0.35–1.51)
No (Ref.)	1	1
**Antenatal care**		
**High-risk factors at first consultation**		
Yes	0.65 (0.22–2.11)	2.12 (1.06–6.07)
No (Ref.)	1	11
**High-risk factors during pregnancy**		
Yes	1.57 (0.72–4.68)	0.57 (0.25–1.26)
No (Ref.)	1	1
**Hospitalization during pregnancy**		
Yes	7.70 (2.96–278.20)	23.16 (0.47–61872.87)
No (Ref.)	1	1
**Adequate antenatal care**		
Yes	1.11 (0.66–1.94)	0.92 (0.43–1.86)
No (Ref.)	1	1
**Gestational age at first consultation**	0.97 (0.92–1.02)	0.96 (0.91–1.00)
**Total number of midwifery-led visits**	0.91 (0.80–0.99)	0.97 (0.86–1.07)

IUGR: intrauterine growth restriction. AOR: adjusted odds ratio. Multivariable logistic regression with Firth’s bias reduction was conducted per parity, adjusting for demographic, social, obstetrical, clinical and antenatal care characteristics.

## DISCUSSION

In this study, we identified factors in pregnant women who received midwifery care that were associated with unplanned visits to emergency services. For nulliparous women who received midwifery care during pregnancy, the odds of visiting spontaneously emergency services during pregnancy were higher in women with more previous pregnancies (i.e. abortion or miscarriage), those without than with high-level hypertension, and those with less previous midwifery-led visits. For multiparous women who received midwifery care during pregnancy, the odds of visiting emergency services were more likely in women presenting factors associated with adverse outcomes at first consultation (i.e. ‘high-risk’ pregnancy) than those without such factors.

For nulliparous women, the odds of visiting emergency services at least once during pregnancy were more likely in nulliparous women with more previous pregnancies compared to nulliparous women with less previous pregnancies. Therefore, a history of a previously lost pregnancy for nulliparous women may lead to an increased need for care during the following pregnancy. According to the report from Europeristat^1^, in the European Union more than 5 million women give birth each year, and another 2 million have a spontaneous or induced abortion (including molar and ectopic pregnancies). Specific data in Belgium are not mentioned; however, a higher number of visits to emergency services during pregnancy in women who previously lost pregnancy can be expected.

For nulliparous women, there was a statistically significant association between emergency visits and hypertension: the odds of visiting emergency services during pregnancy were more likely in women without than those with high-level hypertension. This can be explained by the fact that women with hypertension (i.e. ‘high-risk’ pregnancy) might receive more regular scheduled ANC than women without hypertension, and they might be induced around 38 weeks of gestational age, so they do not attend emergency visits at the very end of the pregnancy. Nevertheless, women’s perception of emergency drives contacts to emergency healthcare services, and high symptom distress or an important discomfort from the specific symptom are associated with increased rates of contacts in healthcare services^19^, not necessarily linked to a sign of severity.

Moreover, for nulliparous women, the odds of visiting emergency services during pregnancy were more likely in women with fewer previous midwifery-led visits than women with more previous midwifery-led visits. Indeed, this supports the idea that, for nulliparous women, visits to emergency services can be a remedy for the lack of access to planned ANC. As underlined by Symon et al.^7^, there is a lack of tailored care to women’s circumstances and needs. The care might not be organized in a manner that offers available and acceptable care to women, as proposed by Renfrew et al.^4^. Moreover, approximately one in five of nulliparae in our sample live in a very precarious situation or without insurance coverage. This high rate is comparable to studies conducted in the same region^17^. These results could uphold the view that women in marginalized groups do not attend their scheduled antenatal appointments, as demonstrated by Downe et al.^25^, but seek care in emergency services when they perceive that they need it.

For multiparous women, the odds of visiting emergency services during pregnancy were more likely in women presenting ‘high-risk’ factors at first consultation than women without such factors. Indeed, multiparous women with pre-existing factors associated with adverse perinatal outcomes might be more likely to feel the need to reach emergency services during pregnancy. In our study, women with ‘high-risk’ factors received obstetric-led care (i.e. ‘Level 3 ’ care) and midwifery care for some antenatal contacts. Indeed, some ‘high-risk’ factors (e.g. multiple miscarriages, conization, genital mutilation) do not require obstetric-led care at every step of the pregnancy, and a midwifery approach to ANC can be beneficial. This can be linked to the POPPIE study testing if a midwifery-led continuity model of care combined with rapid referral to an obstetrician for women at increased risk of preterm birth, improves the experience, perinatal outcomes and quality of care^20^.

Most studies use a stratified risk concept like ours. While we would have preferred to use a more ‘health-oriented’concept as an alternative to ‘risk’ in our study, this was inevitable given the approach adopted by many studies in the current literature. We suggest, similar to Symon et al.^9^, using the terms ‘healthy women and babies with unfavorable health factors of low level’ and ‘women and babies with complications with unfavorable health factors of medium/high/extremely high level’ than referring to high- and low-risk criteria. The great diversity of this sample in terms of maternal nationality (70 nationalities represented) and social status (16% in a very precarious situation) allows for the collection and study of wide and varied data. Moreover, in the universal healthcare system in Belgium^32^, the public status of the healthcare setting allows for the study of ANC provision and access to women in very precarious situations, with low income and/or without health insurance. Including women with adverse social determinants allows the study of perinatal health determinants and access to ANC^1^.

### Limitations

Our study has limitations. For some variables, such as legal civil status, suspected oligoamnios, and hospitalization during pregnancy, the resulting regression coefficients have particularly wide confidence intervals. For these variables, interpretation must be cautious (too wide CIs reduce our confidence in the results). While some degree of collinearity may be expected in both models for including many characteristics, the separation issue alongside considerable imbalance in the levels of many factors (e.g. ‘actual civil status’) may be the main contributors to the wide 95% confidence intervals for many characteristics (Table 2). Separation issues remained even after applying Firth’s correction, hence the wide 95% confidence intervals and the difficulty in finding statistically significant results.

Furthermore, we acknowledge that the sampling method may not provide good external validity, as convenience sampling makes generalization challenging. However, as only one researcher extracted the data, the method chosen was the most efficient and feasible. In addition, the main care leader was not always clearly stated, and women who received only one visit by a midwife were also included. Moreover, data on continuity of care and/or carer were not reported. The results found have to be interpreted carefully. A further study examining continuity of care would allow investigating the association between continuity midwiferyled ANC and visits to emergency healthcare services.

## CONCLUSIONS

Some characteristics seem to be associated with unplanned antenatal visits to emergency services. For nulliparae, the odds of visiting emergency services during pregnancy were more likely in women with more previous pregnancies (i.e. abortion or miscarriage), women without hypertension, and those with fewer previous midwifery-led visits. For multiparae, the odds of visiting emergency services during pregnancy were more likely in women presenting risk factors at the first consultation. Spontaneous visits in emergency services may be driven by a need for care perceived by women and/or their partners, but not specifically by urgent or unfavorable medical conditions. The use of emergency healthcare services can be viewed as a remedy for women with unattended and fragmented planned ANC or with lack of access to it (e.g. women who are members of marginalized population groups^17^). Healthcare settings and providers need to implement a more women-centered care approach, acknowledge diversity and provide care according to women’s actual needs and expectations.

## Data Availability

The data for this study are not available for privacy reasons.
